# Managers’ strategies in handling the COVID-19 pandemic in Norwegian nursing homes and homecare services

**DOI:** 10.1108/LHS-05-2022-0052

**Published:** 2022-11-29

**Authors:** Eline Ree, Siri Wiig, Camilla Seljemo, Torunn Wibe, Hilda Bø Lyng

**Affiliations:** SHARE Centre for Resilience in Healthcare, Department of Quality and Health Technology, Faculty of Health Sciences, University of Stavanger, Stavanger, Norway; Centre for Development of Institutional and Home Care Services, Oslo, Norway; SHARE Centre for Resilience in Healthcare, Department of Quality and Health Technology, Faculty of Health Sciences, University of Stavanger, Stavanger, Norway

**Keywords:** Managers, Home care services, Nursing home, Adaptation, Strategies, Resilience, Pandemic, Home care, Leadership, Elder care, Management, Health and safety, Crisis leadership

## Abstract

**Purpose:**

This study aims to explore nursing home and home care managers’ strategies in handling the COVID-19 pandemic.

**Design/methodology/approach:**

This study has a qualitative design with semistructured individual interviews conducted digitally by videophone (Zoom). Eight managers from nursing homes and five managers from home care services located in a large urban municipality in eastern Norway participated. Systematic text condensation methodology was used for the analysis.

**Findings:**

The managers used several strategies to handle challenges related to the COVID-19 pandemic, including being proactive and thinking ahead in terms of possible scenarios that might occur, continuously training of staff in new procedures and routines and systematic information sharing at all levels, as well as providing different ways of disseminating information for staff, service users and next-of-kins. To handle staffing challenges, managers used strategies such as hiring short-term staff that were temporary laid off from other industries and bringing in students.

**Originality/value:**

The COVID-19 pandemic heavily affected health-care systems worldwide, which has led to many health-care studies. The situation in nursing homes and home care services, which were strongly impacted by the pandemic and in charge of a vulnerable group of people, has not yet received enough attention in research. This study, therefore, seeks to contribute to this research gap by investigating how managers in nursing homes and home care services used different strategies to handle the COVID-19 pandemic.

## Introduction

### Background

Health-care organizations worldwide have experienced a range of acute and more prolonged challenges at the start of and during the COVID-19 pandemic, such as lack of infection control equipment, staff shortages and financial losses (Kaye *et al.*, 2021; Koontalay *et al.*, 2021; Turale *et al.*, 2020). Health-care workers commonly experienced exhaustion and burnout because of being in a new and stressful situation, with continuously changing procedures and routines, lack of staff and high work pressure (Billings *et al.*, 2021; Galanis *et al.*, 2021; Liu *et al.*, 2020). Several studies report worsened physical and mental health for health-care workers and patients due to the pandemic (Chee, 2020; Hugelius *et al.*, 2021; Vindegaard and Benros, 2020). Health-care managers play an important role for the daily quality and safety work and for creating a sound patient safety culture (Johannessen *et al.*, 2020, 2021; Ree and Wiig, 2019). Several studies have found that leadership strategies and styles predict several health-care quality and patient safety outcomes (Boamah *et al.*, 2018; Wong *et al.*, 2013). During the COVID-19 pandemic, health-care managers have a responsibility in dealing with the challenges posed by the pandemic to maintain high-quality care despite being in a crisis (Barasa *et al.*, 2018; Lyng *et al.*, 2021; Teo *et al.*, 2017). This might be particularly challenging in the primary care setting, such as in nursing homes and home care services, as service users in these settings represent a particularly vulnerable group, with the great majority being elderly people at high risk of adverse outcomes in cases of infection.

Although a sudden crisis, such as the COVID-19 pandemic, impose increased risk for errors and adverse events, it can also be an opportunity for development, learning and growth (Wiig *et al.*, 2020). It requires that health-care organizations adapt rapidly to the situation in which managers play an important role (Guise *et al.*, 2014; Ree *et al.*, 2021). In such situations, what exists of protocols and plans will be implemented, at the same time as there will be a need for local changes that must take place quickly (NOU, 2021, p. 6). Crises, such as pandemics, make it necessary for health-care managers to solve new tasks in completely new ways (Bessant *et al.*, 2015; Teo *et al.*, 2017). Several studies show that the managers strategies and behaviors play a key role for how well organizations perform and adapt to challenging or new situations (Kaplan *et al.*, 2010; Taylor *et al.*, 2015; Teo *et al.*, 2017; Paixão *et al.*, 2020; Sriharan *et al.*, 2022). For example, a systematic review by Taylor *et al.* (2015) found that high-performing hospitals were characterized by having senior management that was visible, inclusive and available for staff, as well as being committed to high-quality care. A recently published scoping review identified several leadership competencies that were key to manage pandemic responses (Sriharan *et al.*, 2022). These included strategies such as preparing, planning, and effective communication and collaboration, engage and inspire others and the ability to adapt rapidly to new or changing situations. The study calls for more research on pandemic leadership (Sriharan *et al.*, 2022). The current study seeks to add knowledge of what strategies health-care managers in nursing homes and home care services use to adapt to the COVID-19 situation, including the development of new innovative work practices, methods and procedures, to best take care of service users.

The resilience perspective is highlighted as valuable for understanding how organizations adapt to challenges, changes and risk, and how organizations succeed in performing their tasks even if exposed to pressure and strain, such as a pandemic (Wiig *et al.*, 2020). Based on Hollnagel’s (2018) description of resilience potentials and Berg and Aase’s (2019) suggestions of cognitive and behavioral strategies that characterize resilience in health-care organizations, Ree *et al.* (2021) have proposed a model of managers’ role in supporting resilience. They argue that:

Managers’ role in supporting resilience consists of the strategies managers use to engage people in collaborative and coordinated processes that adapt, enhance or reorganize system functioning, promoting possibilities for learning, growth, development and recovery of the healthcare system to maintain high quality care (Ree *et al.*, 2021).

This approach will be used to guide the current study. The model by Ree *et al.* (2021) draws on examples and experiences from managers’ everyday practice in nursing homes and home care, and it will therefore be of interest to explore whether and how this model can be applied in a crisis setting, such as the COVID-19 pandemic. Similarities between management strategies during everyday operations and management strategies during crisis can be understood through Kruk *et al.* (2017) argument, which states that the ability of systems to perform well during chronic stress often also leads to an ability to perform well during situations of shocks and crisis.

Another stream of management literature important for understanding management strategies in health-care systems is the complexity leadership perspective (Hazy and Uhl-Bien, 2015; Marion and Uhl-Bien, 2001; Uhl-Bien and Arena, 2018; Uhl-Bien *et al.*, 2007). Hazy and Uhl-Bien (2015) describe five leadership strategies of importance for complexity leadership:

1. generative functions (e.g. adopting innovations and try new things);
2. administrative functions (e.g. good planning and organizing to meet goals);
3. community-building functions (e.g. involve staff and building trust);
4. information gathering functions (e.g. encourage collaboration across teams/organization, identify and test successful initiatives); and
5. information using functions (e.g. delegate and clarify responsibility to staff members).

Findings from this study will therefore be discussed in relation to both these leadership approaches to provide a broader understanding of the findings. Uhl-Bien *et al.* (2007) propose that leadership should be seen as:

Not only as position and authority but also as an emergent, interactive dynamic – a complex interplay from which a collective impetus for action and change emerges when heterogeneous agents interact in networks in ways that produce new patterns of behavior or new modes of operating.

Although the current study includes managers having formally assigned management roles for health-care personnel and/or professional tasks, they might use different more or less successful leadership strategies in dealing with challenging situations, such as the COVID-19 pandemic.

Although several previous studies explore challenges introduced by the COVID-19 pandemic to the health-care system, fewer studies address the strategies used by managers to overcome the obstacles faced by the pandemic and manage in a continuously changing situation. Furthermore, the primary health-care setting of managers’ strategies in nursing homes and home care services represents a gap in the literature. This study contributes new understanding of managers’ strategies for handling early phases of the COVID-19 pandemic in nursing homes and home care services.

### Aim and research questions

The aim of this study is to explore nursing home and home care managers’ strategies of handling the COVID-19 pandemic. This aim was explored through the following research questions:

RQ1. What type of strategies did managers use to handle the challenges faced during the pandemic?

RQ2. How did managers continuously manage and integrate new knowledge and information during the pandemic to ensure high-quality care?

## Methods

The study has an explorative qualitative design.

### Setting

The study setting is nursing homes and home care services located in a large (in population size) urban municipality in eastern Norway. In Norway, nursing homes and home care services are delivered by the municipalities, in line with other primary health-care services such as general practitioners, after-hours emergency services, midwife, physiotherapy and rehabilitation. The availability of and access to primary care services vary across different municipalities, often due to geographical location and size of the municipality (Johannessen *et al.*, 2020; Ree *et al.*, 2019). The home care services consist of health-care services provided in the service users own homes to individuals who are disabled or ill, such as assistance with personal hygiene, medication monitoring and/or assistance and wound treatment. The number of service users in the home care services is varying from time to time, but normally there are approximately 200 service users per department (see Table 1 for number of departments in each home care included in this study). A department manager has responsibility for ± 80 employees. Nursing homes are institutions with health-care staff present 24 h a day to care for people who are too ill or disabled to being cared for at home. The size including number of department varies across the included nursing homes (see Table 1), but a department manager normally has responsibility for ± 30 employees. The municipality included in the current study is central, including a large city and short distances between primary and specialist health-care services. It is small when it comes to geographical size. The home care services and nursing homes included do, however, vary in size, and during the COVID-19 pandemic, the infection pressure varied across the different units, resulting in some variations in challenges and strategies used by managers to handle the situation. The Norwegian Government implemented extensive countrywide restrictions 12th of March 2020, including the closing of educational institutions, cultural events and the food service industry. In the following days, most health and welfare services were also shut down, except the most critical ones. There were extensive restrictions in the health-care sector, and rules about the acquisition and use of infection control equipment. In nursing homes, visitors were generally not allowed, and the public were encouraged to stay at home and not physically meet people outside their household, especially not vulnerable people such as elderly and chronically sick individuals. Staff in home care services should seek to provide remote care as far as possible, which resulted in many elderlies being isolated home alone.

### Recruitment and participants

One of the authors (TW), a consultant at the Center for Development of Institutional and Home care Services (USHT), had the role as a key contact, helping recruit the units by contacting the top managers of relevant units in eastern Norway. A convenience sample of 13 female managers from eight nursing homes and five home care services were recruited (see Table 1). All managers were incumbents at the time of pandemic being declared by the World Health Organization. The managerial levels spanned from nursing home and home care managers with an overall managerial responsibility of the units (*n* = 1), department managers with personnel responsibility for different departments within the units (*n* = 7), quality managers (*n* = 4) and professional development nurse (*n* = 1). Sample size was guided by the assessment of information power, considering the specificity of the research question and sample, the use of theory, the quality of the interviews and the analysis strategy (Malterud *et al.*, 2016). Specific research questions were developed to guide sample selection, interviews and the analysis process. Furthermore, the sample was specific and relevant for the study aim, all participants provided rich information relevant for the research questions, and theoretical frameworks were used to support the study. Although a thematic cross-case analysis strategy was used (see analysis section below), we considered that the 13 interviews provided sufficient information power to illuminate the research questions (Malterud *et al.*, 2016).

### Data collection

Individual semistructured interviews were conducted by researchers in the project team from the end of September to November 2020. Due to restrictions following the COVID-19 pandemic, all interviews were conducted via telephone or Zoom, and lasted between 30 and 60 min. The interviews were audiotaped and transcribed verbatim. The interview guide contained questions about managers’ strategies for handling the COVID-19 pandemic. The guide covered topics such as leadership practices, perceived success factors and barriers, integration of new knowledge and information, as well as development or implementation of new and innovative solutions during the pandemic.

### Analysis

The data material was analyzed in a four-step process using systematic text condensation, a thematic cross-case analysis strategy (Malterud, 2012). In step one, the first author (ER) read through the transcripts to get an overall impression of the data material, and established preliminary themes related to managers strategies used to handle the pandemic. Examples of preliminary themes were staffing challenges, emergency preparedness, availability/presence of managers, cooperation and togetherness and education and training in new procedures. The content and meaning of these themes were then discussed with three of the coauthors (SW, HBL and CS), resulting in adjustments in some of the theme names. In step two, ER identified meaning units related to the research questions and coded them in relation to the preliminary themes resulting in code groups describing managers strategies. During the process of establishing code groups, some preliminary themes were renamed, some were split because they covered different issues and some were lumped because they represented aspects of the same issue. In step three, the content in each code group were condensed and sorted into subgroups. In the fourth and final step, the content of the condensates was synthesized to provide a meaningful analytic text about managers’ experiences and strategies of handling the COVID-19 pandemic.

## Results

Box. Summary of main challenges faced by the managers during the COVID-19 pandemic:

The main challenges faced during the pandemic were related to insufficient contingency plans and infection control, lack of staffing, constantly changing guidelines and routines and challenges due to communication and information flow.

The analysis revealed several strategies and adaptations used by the managers to handle the challenges of the COVID-19 pandemic. The managers emphasized the importance of being proactive and thinking ahead about possible scenarios that might occur, systematic information sharing at all levels and continuously training of staff in new procedures and routines. The managers encouraged togetherness and collaboration within and across units and being available and present as managers to secure a good psychosocial work environment during the pandemic. To handle staffing challenges and ensure good care, managers used strategies such as hiring temporary staff and students and finding new ways of organizing work and activities to make everyday life meaningful to the service users. These findings are elaborated in the following.

### Being proactive and finding new ways of working to adapt to the situation

When the COVID-19 pandemic first hit, some of the managers experienced being already partly prepared because they had witnessed what happened in China and other countries. A good deal of work had been done in advance in some of the nursing homes and home care services. For example, they had developed a contingency plan, completed a risk- and vulnerability analysis, made plans on where to put up isolates and rooms with clean and unclean zones if needed, as well as having several general infection control routines in place. Those who already were partly prepared experienced that the job was much easier when the pandemic first arrived.

The managers talked about the importance of always be proactive and thinking ahead about possible scenarios that might occur. Being prepared, both before and after the first outbreak of the pandemic, was significant for how well the managers experienced to handle the situation. This ranged from obvious important strategies like having contingency plans and appropriate equipment, to smaller strategies like having updated lists of telephone numbers for next-of-kins. Some of the managers anticipated the struggle between institutions and even between countries regarding infection control equipment before the pandemic came to Norway, and therefore procured reserves such as extra infection coats, face masks and hand sanitizers early on. The managers said they were more prepared today, as they had learned from what happened during the first outbreak both globally, national and in their own units, and thus created scenarios for various situations that might occur in the future. If, for example, an employee is ill, a manager said, he now knew that he had to map those he/she had interacted with. Several units were quick to create contingency teams, which communicated closely with the managers. As illustrated by the following quote, the contingency team knew what tasks they were to perform, and could show up at short notice and get an overview if a situation occurred that required action:

I think the most important thing is to have a contingency team. That is, who knows what tasks to perform when we get a case. That you locate the infection control equipment, mark the department’s clean and unclean zones, provide information to employees, and ensure infection detection. That each and everyone has their assigned tasks, I think that is important. (Quality manager, nursing home).

The managers also adapted by coming up with new ways of working. For example, they cleaned the rooms in the nursing homes in a different way, using their own staff. They went over and washed all desks in use, twice a day, as well as having the cleaning personnel doing more thorough cleaning regularly. They had infection control trolleys where they had all they needed to enter rooms with infected users, such as gloves, garbage bags, face masks, paper, alcohol and glasses, as well as having posters visible in all rooms that stated what to do, for example, when entering a room with infection.

Several emphasized the relief they felt when they received a newly developed infection tracking tool, which was a computer program that made it easier for them to get an overview of and track close contacts. The tool made their work a lot easier since it was used by the whole municipality and, therefore, allowed them to transfer patients between different health-care services. Previous of the software, they had to spend a lot of time calling around and making endless lists of infected patients. Other examples of managerial strategies where having updated lists of service users who were admitted and discharged, having enough staff available if something happened in the departments, and having an overview of service users that could share rooms. An example from the home care services was more use of remote care, such as digitally helping users with medicine dispensers and weight control:

Before, they handed out […] for example, medicine for 14 days. But then you still had to stop by on each visit, to deliver that medicine. But then they have found out, sort of, there is a lot of technology. That they can actually handle this themselves. And thus, minimize visits (Professional development nurse, home care services)

### Information dissemination, education and training in new procedures and routines

During the pandemic, the managers became more systematic in spreading information at all levels, as well as disseminate information in different ways to reach everyone, from staff to service users and next-of-kins. To prevent the amount of new information from being overwhelming, as well as ensuring that everyone understood the new procedures in the same way, the managers compressed the information as much as possible and used a comprehensible language adapted to the receivers. They then presented the information to their employees regularly at department meetings, as well as spreading information by text messages on mobile and e-mail and/or a weekly Newsletter. These actions were not necessarily new, but they did it more systematically and regularly due to the continuously changing situation and the increased amount of information needed to be disseminated to staff. The managers created their own interdisciplinary corona team consisting of representatives from the workforce, such as registered and professional nurses, other health professionals and unskilled workers, who were given the responsibility of spreading new information. Involvement of staff and ensuring consistency in information disseminated by different actors (i.e. department managers, professional nurses and quality managers) was key, and helped avoid information failure.

The managers said that the COVID-19 pandemic had led to the development of new tools, technology and new ways of working, many in which they planned to continue using when the pandemic was over. For example, they showed film clips, both from YouTube and the Competence Bridge (a digital platform for coordination and competence sharing between municipalities, hospitals and educational institutions). Gradually, most of them used a digital tool called Workplace by Facebook to share new information. The tool works like a “business version” of Facebook where managers could post information and monitor by whom and when the information was read. Digital tools were found to work well in information dissemination. Managers also increased their use of online management team meetings, which was considered time-saving. They became more aware of what meetings and activities to prioritize, which made them more efficient. Being efficient was also necessary in the information work, as illustrated by this quote:

It has been difficult for me to reach out to my employees. So, what I have done, is to have small 15-minute meetings where I just enter and provide the information and ask if there is anything they are wondering about. I have had such meetings often in some periods because it is a way to reach many (department manager, home care services).

The managers found it important to repeatedly train employees in new and continuously changing procedures, and much more effort was directed to training than before the pandemic. They trained a group of nurses as infection nurses to be resource persons helping them educate and train the staff. These nurses were drilled in infection control. They worked closely with the managers, were always present among staff and were responsible of talking about infection, training and testing. Involving staff in teaching and information work, was perceived as an important success strategy by the managers, contributing to more engagement and motivation in staff and easier adoption of new information and procedures. Some managers experienced that several employees actually flourished during the pandemic, as they became aware of the knowledge they actually had or that they learned new skills and new ways to use situated knowledge.

At all units, they had regularly education and training of infection control routines at the work site. They also had several e-learning courses about infection control routines. In some units, they divided staff in smaller groups and showed films about, for example, hand hygiene or practiced scenarios. Practicing scenarios was perceived as essential for being prepared and knowing exactly what to do when the situation changed or something happened. They used visualization and simulation techniques to a much greater extent than before the pandemic to learn what to do in real situations, for example, in the service users’ own homes. A quality manager gave an example of how they practiced at their department:

We have had regular drills in the departments, weekly. Drills in relation to dressing and undressing. And when we get an outbreak or a case, we have a dressing and undressing guard who overlooks that everyone is doing the right things (quality manager, nursing home).

### The managers encouraged togetherness and being available and present as managers to secure a good psychosocial work environment during the pandemic

The managers emphasized the importance of working together to handle the impacts of the pandemic and that this pronounced effort led to increased collaboration both within and across units, between professional groups and between managers and staff. The managers had the perception that employees felt more needed than before the pandemic, as they realized it was necessary for everyone to work together and contribute to overcome the obstacles faced. This resulted in staff across different departments and services got to know each other, which again created a strong feeling of unity and team cohesion among the employees, as well as a greater flexibility and understanding of the necessity of everyone’s roles. Trusting and helping each other and being aware that everyone was in this together seemed to be one of the most important strategies in dealing with the pandemic, according to the managers.

The managers often praised the employees and gave them positive feedback. Although there were areas of improvement, the managers found it important to focus on what the employees succeeded with and what worked well and discussed with employees how they could use these positive experiences to succeed in other situations as well.

Better collaboration and increased team cohesion applied for the management groups as well. During the pandemic, management groups across departments in nursing homes and home care had daily online meetings where they shared information about current status, routines and equipment and discussed matters they were uncertain about. Mutual trust, collaboration and common goals within and across management groups were highlighted as keys to succeed in dealing with the pandemic. A manager emphasized the collaboration within the management team as particularly positive as it led to a closer connection between the managers:

Everyone worked together on this. So, there was a better collegiality, so to speak. And I think that’s very important, that we see that we’re in this together (department manager, nursing home).

Being present as managers, both for staff, next-of-kin and service users, ensuring that everyone felt seen and heard, was perceived as key for successful handling of the pandemic. One of the managers said they provided a kind of psychological support, which was particularly important in a situation that was new to everyone, unpredictable and demanding. The managers spend a lot of time “at the floor,” trying to meet up with as many of their employees as possible during the workday. Even though the authorities recommended using home office as much as possible, all the managers stated that they found it more important being present at work contributing to the psychosocial work environment during these challenging times.

When it was impossible to be present, managers practiced a lot of distance follow-up, which was a good alternative since it was less resource-intensive, making it easier to reach more people. Many of the managers had made it clear to their employees that there was a low threshold for calling them, even in the weekends. Being available and updated at all times was extremely demanding, and required that the managers had great work capacity, little rigidity, an ability of not being preoccupied with restrictions regarding their own working hours, and good communication skills with both employees, service users and next-of-kins. The following quote illustrates the importance of manager presence:

There was a lot of talk about home office, but I had very little of it. Because there was almost a queue outside my office all the time. People always had something to ask about. So, I think it was important to be a manager that was present (department manager, nursing home).

### Strategies and adaptations to ensure proper staffing and care for service users during the pandemic

When the pandemic hit, many units first tried to manage with the resources they had in the department, increasing the number of extra shifts and borrowing extra personnel from other departments when possible. The units that were best prepared before the pandemic hit already had a collaboration plan across departments. Some of the units were lucky because there were more students than normally, with different backgrounds, such as medical students, nursing students and other health-care students available to work ordinary paid shifts in addition to the student practice due to the lockdown at university campuses. Other units had to be more creative in their search for staff during the pandemic. For example, one of the nursing homes had over 40 employees in quarantine at some point and used all sorts of media, such as Facebook and Instagram, to communicate their search for staff. They then experienced that a lot of people who were temporary laid off from different industries, such as restaurants, the hotel industry and photography, showed interest to work at their nursing home. Despite not having health-care related backgrounds, these people were considered very useful since they possessed good people skills, and contributed with cooking, food serving and talking with the service users.

During the pandemic, some of the low threshold services were reduced or shut down in some nursing homes due to strict infection control measures. Managers responded to this by reallocating staff from these services to other departments within the unit to avoid layoffs. They made brand-new shifts to use the staff in a more efficient way, as well as increasing the work percentage of many positions. This was necessary strategies since several nurses and health-care workers were assigned to work in contingency teams with pandemic-specific issues. Many talked about the importance of being flexible, as exemplified by one of the managers:

I see that we needed to be very flexible considering that we have had both service users and employees in quarantine and isolation. And we had to look up and see more across the sections. So, in that way we have used resources more appropriately (department manager, home care services).

When the pandemic hit, the managers went through the scope of health-care services provided to the service users to assess what were absolutely necessary to retain if, for example, half of the workforce disappeared. They then realized that they could cut in many of the services, implying that they had not been good enough to evaluate the services before. They started to follow-up several service users by phone, and after a while, it turned out that many of them managed more on their own and needed less health services than previously received.

Managers at nursing homes had to think differently about how to make everyday life meaningful to the service users. Normally they used specific cultural leaders and music therapists to facilitate activities for them, but with less personnel available, those present at work had to arrange for activities with the service users. Furthermore, the managers emphasized the importance of having a good dialogue with next-of-kins, with frequent updates on the situation and having a pragmatic attitude toward the strict visiting rules. A manager said that one of the most important things they did during the pandemic was to facilitate contact between the service users and their loved ones. The managers strived to adapt the rules from the authorities to the individual service user. Thus, even though visits were not allowed at the nursing home, they still made it happen in a safe environment. The managers emphasized the importance of relying on their own competence and ability for reflection and assessment when adjusting some of the rules to get things going and take care of their service users during the pandemic. As expressed by the top manager at a nursing home:

We have to make individual adjustments in the situation we are in now. Where we do a kind of cognitive situation-specific risk analysis for each service user who struggles. Or for every next-of-kin it is difficult for (Head of nursing home).

## Discussion

**Figure 1.**
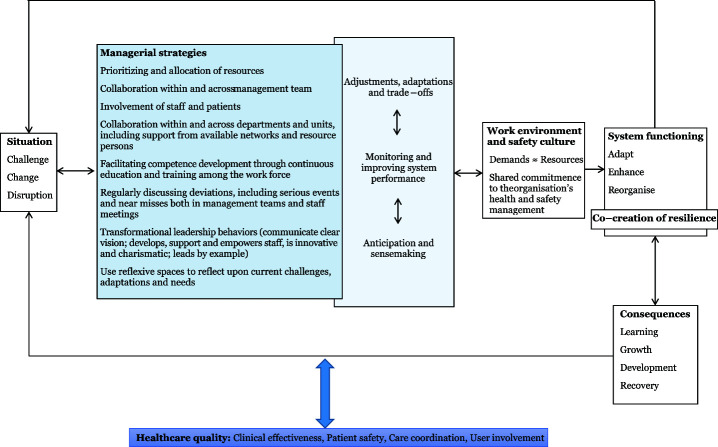
A model of managers' role in supporting resilience (Ree *et al.*, 2021)

This novel study addresses managers’ strategies in dealing with the early phase of the COVID-19 pandemic in Norwegian nursing homes and home care services. Findings showed that the managers used specific strategies related to the following categories to ensure resilient care for service users: different ways of adapting to the situation, disseminating information, provide training in new procedures, ensuring a good psychosocial work environment, and to provide proper staffing, see Table 2 for the specific strategies used. In the following, the findings from the current study will be discussed in relation to Ree *et al.* (2021) proposed model of managers’ role in supporting resilience (see Figure 1), and complexity leadership theory (Hazy and Uhl-Bien, 2015) to provide an understanding of our findings in terms of different perspectives of relevance for leadership in health care.

The need for adaptive capacity is a key aspect both within the resilience in health-care literature and the complexity leadership literature. However, managers play a key role for strengthening adaptive capacity within the organization (Hazy and Uhl-Bien, 2015; Lyng *et al.*, 2022; Ree *et al.*, 2021; Teo *et al.*, 2017). Hazy and Uhl-Bien (2015) state that generative functions in leadership facilitate adaptation, entrepreneurial activities and experimentation. As such, managers need an openness to new perspectives, encourage the adoption of new products and procedures and allow for bottom-up initiatives. Involving staff in a bottom-up approach was also found to be a valuable strategy when seeking resilient performance (Ree *et al.*, 2021) and was an important strategy in the current study to meet the challenges posed by the COVID-19 pandemic.

An important part of leadership is to make trade-offs between demands and capacity (Anderson *et al.*, 2016; Anderson *et al.*, 2020; Ree *et al.*, 2021). The managers informing this study where always in need of making trade-offs across the macro (government guidelines) and micro level (frontline needs). In terms of COVID-19, these trade-offs consisted of demands and guidelines of how to provide care during a pandemic, with an often restricted capacity due to quarantine and infection among staff. Efforts to align demands and capacity are not efforts specifically linked to the management of pandemics, and it is a key task in all parts of leadership (Ree *et al.*, 2021). However, during the pandemic, the need for managers to make rapid trade-offs under high levels of uncertainty were more critical. Trade-offs can therefore be considered a part of administrative leadership, where available resources are distributed to ensure efficiency and quality performance (Hazy and Uhl-Bien, 2015). As such, trade-offs where convergent actions seeking to establish dynamic stability in times of high uncertainty, like when managers ensured contact between next-of-kin and service users.

Administrative leadership was found to include important strategies to improve system performance. These efforts included reorganization and reevaluation of resources, like when managers engaged in basic training and teaching of staff to ensure compliance to procedures and guidelines and when evaluating the appropriate level of remote care for each service user. As such, administrative leadership ensured patterns of action as a response to macrolevel demands (Hazy and Uhl-Bien, 2015).

Strategies for monitoring were important actions to keep up with the continuous amount of COVID-19 information, service users’ movements and the availability of staff. These forms of strategies align with the information-gathering function described by Hazy and Uhl-Bien (2015). Information-gathering strategies include activities like observation, exploring the environment and information transfer within the organization (Hazy and Uhl-Bien, 2015). In terms of COVID-19, the extensive amount of information provided to managers introduced a need for filtering the information to be further disseminated to the staff.

Contextual understanding is an important precursor for anticipation (Øyri and Wiig, 2022). Having such understanding, gathered through monitoring actions of front-line practice, ensured an ability to prepare proactive responses that would ease upcoming situations. In terms of COVID-19, information-using strategies for anticipation included infection-control plans, procedures and equipment. Additionally, information-using strategies were also found important for sensemaking. Managers had to identify ways of disseminating the continuous flow of information, translate the information to make it easier accessible for staff and filter out information that was not important to disseminate. Furthermore, strict guidelines from the macrolevel needed to be contextualized and individualized, based on the understanding of the individual service user, physical environment and available resources.

To provide safety to staff at work, managers stated the need for being available and present. Even though home office was recommended for all administrative personnel, health-care managers found it necessary to be present at the front-line to make the staff feel safer. Additionally, managers from different organizations engaged in frequent meetings, which provided support to the managers and a feeling of being part of a community. These strategies, therefore, align with what Hazy and Uhl-Bien (2015) describe as community-building functions, which highlight the sense of belonging and shared objectives. A strategy for ensuring shared objectives found in our analysis was the promotion and spread of successful actions within the organization.

Throughout this discussion, we have found a correspondence between resilient leadership strategies (Ree *et al.*, 2021) and complexity leadership functions (Hazy and Uhl-Bien, 2015). As such, a theoretical bridge between these two streams of literature has been illustrated. The results further illustrate the important role of managers in rapidly adapting to the new and continuously changing COVID-19 situation.

## Limitations

This qualitative study has some limitations that should be acknowledged. First, the number of participants is limited. However, based on their information power, being nursing home and home care managers in the most heavily COVID-19 affected area in Norway, they shared comprehensive and rich information. Second, the data for this study was collected 6–9 months after the COVID-19 pandemic outbreak in Norway. This delay in data collection may influence the results as the managers felt they had “survived” the first wave and felt more relaxed based on their new knowledge and experience. Third, there might be a risk for social desirability bias where the managers present themselves and their units/departments in a favorable way. We strived to limit the possibility of such biases during the interviews by asking for concrete examples of what the managers did and thorough descriptions of their applied strategies.

## Conclusions and implications

This paper explores managers’ strategies for handling the COVID-19 pandemic in Norwegian nursing homes and home care services. The results show that all managers had to use new and creative strategies to deal with the challenges faced during the COVID-19 pandemic. Identified strategies of value in handling the pandemic included: being proactive in planning, facilitate a necessary room for adaptions to the situation, provide different ways of disseminating new knowledge and information, ensure training in new procedures, present leadership, ensuring a good psychosocial work environment and involvement of staff and to establish strategies for proper staffing.

This study contributes new understanding of how managers in nursing homes and home care services used different strategies in their effort to ensure quality in care for their service users in a challenging situation. Such knowledge can be useful for future crisis in the health-care sector, such as new pandemics or outbreaks, but also for more or less planned changes, such as restructuring or merges and in the daily work of managers. We recommend policymakers, educational institutions and leadership development to acknowledge and integrate these aspects into diverse types of leadership expectations, course curriculum and training programs.

The study displays a relatedness between literature on resilient leadership and complexity leadership. Theoretical implications are as such, bridging of theories of resilient leadership and complexity leadership, and the use of these theories for exploring managerial strategies in a pandemic setting in Norway. Future studies are needed to provide an understanding of how findings from this study relate to other national contexts, considering different impacts of COVID-19, population and type of health-care system. Furthermore, as COVID-19 still is a global challenge, we call for new studies to explore managerial strategies in later phases of the pandemic. Studies combining interviews from managers and staff, as well as quantitative studies exploring the relationship between managerial strategies and important organizational outcomes (e.g. patient satisfaction and mortality, staff engagement, staff knowledge about procedures and guidelines, turnover), can contribute a broader picture of the adaptability and effectiveness of managerial strategies in the health-care sector.

## Acknowledgement

The authors would like to thank Lene Schibevaag for contributing to the data collection. The authors would also like to thank the participants for sharing their experiences.

*Funding*: No funding was received for this study. Siri Wiig and Hilda Bø Lyng were supported by the Resilience in health-care program (RiH). The RiH research program has received funding from the Research Council of Norway under the FRIPRO Toppforsk program, grant agreement no 275,367. The University of Stavanger, Norway, the Norwegian University of Science and Technology in Gjøvik, and the Norwegian Air Ambulance Foundation support the program with in-kind funding. The funding bodies played no role in the research process.

*Ethics approval and consent to participate*: The study is supported by the Norwegian Center for Research Data (NSD, ID: 317328). The study followed the principles of the Helsinki declaration, including written informed consent to participate from all participants.

*Availability of data and materials*: The data material used in this study is available upon request from the corresponding author.

*Competing interests*: The authors declare that they have no competing interests.

*Authors’ contributions*: ER, HB and SW came up with the initial idea of the article. TW facilitated the first contact with participating units. HBL, CS and TW conducted the data collection. ER led the analysis in close collaboration with HBL, SW and CS. ER drafted the manuscript with significant contributions from HBL. All authors provided input to study drafts, and all authors read and approved the final manuscript.

## Figures and Tables

**Table 1.** Sample information

	Type of unit	Professional title	Age group	Size of unit	Education/work experience
1	Nursing home	Department manager	51–55	146 residents across five departments	Education: registered nurse Years of experience: not stated (nursing homes and home care) Years in current position: not stated
2	Nursing home	Quality manager	41–45	90 residents across three departments	Education: registered nurse with further education in leadership and welfare technology Years of experience: not stated (nursing homes and home care) Years in current position: not stated
3	Nursing home	Quality manager	46–50	85 residents across five departments	Education: registered nurse with master’s degree in health-care services for elderly Years of experience: 20 (nursing homes) Years in current position: 2.5
4	Home care	Department manager	51–55	Four departments	Education: physiotherapist with further leadership education Years of experience: not stated, but worked several years as leader, also in former positions Years in current position: not specifically stated (a couple of years)
5	Nursing home	Department manager	36–40	140 residents (short-term patients) across six departments	Education: registered nurse with further leadership education Years of experience: not stated (nursing homes) Years in current position: 1
6	Nursing home	Head of nursing home	41–45	96 residents across three departments	Education: registered nurse with further education in mental health care. No formal leadership education, but shorter leadership courses Years of experience: 21 years (hospital and nursing home). Eight years of leader experience Years in current position: 5
7	Home care	Department manager	36–40	140 residents (short-term patients) across six departments	Education: registered nurse with further leadership education Years of experience: 19 (including two years as leader) Years in current position: 2
8	Home care	Department manager	36–40	Four departments	Education: environmental therapist Years of experience: 5 Years in current position: 2
9	Nursing home	Quality manager	41–45	48 residents across two departments	Education: registered nurse and courses in quality leadership Years of experience: not stated, but several years as leader in hospital and in municipal health-care services Years in current position: eight months (since March)
10	Home care	Department manager	Not stated	Three departments	Education: registered nurse with further education in health leadership Years of experience: 20+ (hospital and nursing homes both as nurse and manager) Years in current position: 2
11	Home care	Department manager	46–50	Three departments	Education: registered nurse with master’s degree in leadership Years of experience: total not stated. 11 years as leader (nursing homes and home care services) Years in current position: not stated
12	Nursing home	Quality manager	36–40	132 residents across six departments	Education: registered nurse with further education in leadership Years of experience: 18 (including previous leadership position) Years in current position: one month, but worked as department manager at the same unit before this position
13	Home care	Professional development nurse	46–50	Four departments	Education: Registered nurse Years of experience: 20 years (nursing homes, home care, university sector) Years in current position: 10 months

**Table 2.** Finding’s relations to existing literature of managerial strategies for resilience in health care and complex organizations

Findings from this study	Managerial strategies supporting resilience^a^	Complexity leadership^b^
Adjusting guidelines to local context New procedures and practices New ways of recruiting and organize staff Reorganization of resources	Adaptations/adjustments	Generative leadership
Ensure contact between residents and next-of-kin Leaders prioritized being available for staff Adapting practices to available resources	Trade-offs	Administrative leadership
Used students as buffer resources Training and educating staff Improving cleaning procedures Using new software for information transfer and coordination of care Reevaluate practices, making care more efficient	Improving system performance	Administrative leadership
Constantly updating and disseminating new guidelines Keeping updated of resident’s movements Keeping updated on available staff	Monitoring	Information gathering
Initiate plans before the pandemic hit Norway, based on information from China Developing scenarios as proactive activities Making plans for available resources (staff, infection control equipment) Providing easy access to infection material Establish a COVID team	Anticipation	Information using leadership
Providing access of COVID information to staff Translation of new information to staff to ease understanding Used situated understanding to evaluate risk for each patient	Sensemaking	Information using leadership
Learning from what goes well Frequent interaction among leaders Leaders were present at the front-line Collaboration across departments	Availability and collaboration	Community building

Notes: ^a^Based on Ree *et al.* (2021), ^b^Based on Hazy and Uhl-Bien (2015)
